# Draft Genome Sequence of the Moderately Thermophilic Actinobacterial Steroid-Transforming Saccharopolyspora hirsuta subsp. *hirsuta* Strain VKM Ac-666^T^

**DOI:** 10.1128/MRA.01327-19

**Published:** 2020-01-02

**Authors:** Tatyana G. Lobastova, Victoria V. Fokina, Eugeny Y. Bragin, Victoriya Y. Shtratnikova, Irina P. Starodumova, Sergey V. Tarlachkov, Marina V. Donova

**Affiliations:** a G.K. Skryabin Institute of Biochemistry and Physiology of Microorganisms, Pushchino Scientific Center for Biological Research of the Russian Academy of Sciences, Federal Research Center, Pushchino, Russian Federation; b A.N. Belozersky Research Institute of Physico-Chemical Biology, M.V. Lomonosov Moscow State University, Moscow, Russian Federation; c Branch of Shemyakin and Ovchinnikov Institute of Bioorganic Chemistry, Russian Academy of Sciences, Pushchino, Russian Federation; Portland State University

## Abstract

The draft genome sequence of the type strain Saccharopolyspora hirsuta subsp. *hirsuta* VKM Ac-666 was sequenced. This moderately thermophilic actinobacterial strain of sugarcane bagasse origin is able to transform different steroid substrates.

## ANNOUNCEMENT

Actinobacteria from different habitats were described to utilize steroids as ubiquitous growth substrates or to perform structural modifications of natural or synthetic steroids as a prelude to their further full degradation as a carbon and energy source. The fundamentals of steroid catabolism by actinobacteria have been studied mainly for mesophilic species (1, 2), while little is known of the features of thermophilic actinobacteria capable of steroid oxidation (3, 4).

Thermophilic actinobacteria of Saccharopolyspora hirsuta subsp. *hirsuta* were originally isolated from spontaneously heated sugarcane bagasse (5). The strain utilized lithocholic acid (6), transformed 3β-ol-5-ene steroids to their corresponding 3-keto-4-ene derivatives, and catalyzed 1(2)-dehydrogenation as it was demonstrated for dehydroepiandrosterone and 7-hydroxylated steroid d-lactones (7); however, the molecular mechanisms of its steroid-transforming activities have not been studied yet. Here, we report a whole-genome sequence of the strain.

The strain *S. hirsuta* subsp. *hirsuta* VKM Ac-666^T^ was obtained from the All-Russian Collection of Microorganisms (VKM) of the G.K. Skryabin Institute of Biochemistry and Physiology of Microorganisms at the Russian Academy of Sciences.

Genomic DNA extraction from *S. hirsuta* subsp. *hirsuta* VKM Ac-666^T^ was carried out as described (8) with the following modifications. Cells were lysed with lysozyme, SDS, and proteinase K. RNA was removed by RNase digestion. DNA was purified by phenol extraction and isopropanol precipitation.

Fragmentation of the genomic DNA was done by sonication with a Covaris S220 instrument. The short-read library containing DNA fragments of 300- to 400-bp insert lengths was prepared with a NEBNext Ultra II DNA library prep kit for Illumina. The library was sequenced twice, first on an Illumina HiSeq 4000 platform with a HiSeq 3000/4000 PE cluster kit and a HiSeq 3000/4000 SBS kit (300 cycles) and then on an Illumina HiSeq 2500 platform with a HiSeq rapid PE cluster kit v2 and a HiSeq rapid SBS kit v2 (500 cycles).

Default parameters were used for all software unless otherwise specified. The read quality was checked with FastQC v0.11.8 (9). Raw Illumina sequencing data were adapter trimmed using BBDuk v38.35 (10) (with the parameters ktrim, r; k, 23; mink, 11; hdist, 1; tpe; minlen, 20; and ref, adapters) and then filtered to remove the known Illumina artifacts and PhiX using BBDuk v38.35 (10) (with the parameters k, 31; ref, artifacts,phix; and cardinality). To remove possible contamination, the reads were mapped to masked versions of a human reference genome (hg38) and discarded if the identity exceeded 95% using BBMap v38.35 (10) (with the parameters minid, 0.95; maxindel, 3; bwr, 0.16; bw, 12; quickmatch; fast; and minhits, 2). The remaining reads were trimmed by quality scores using BBDuk v38.35 (10) (with the parameters qtrim, r; trimq, 15; and minlen, 20).

Clean sequencing data were assembled using SPAdes v3.13.0 (11) with the parameters -careful and -cov-cutoff auto, and the resulting contigs were discarded if their length was <500 bp. Genome annotation was performed with the NCBI Prokaryotic Genome Automatic Annotation Pipeline (12). The 16S rRNA gene sequence of strain VKM Ac-666^T^ (MN515057) was obtained using the Sanger sequencing method and compared with the 16S rRNA sequence extracted from the whole-genome assembly to ensure the authenticity of genome data. The pairwise similarity between the 16S rRNA gene sequences was determined using TaxonDC v1.3 (13).

A total of 5,461,137 paired-end reads with a length of 251 bp (2.7 Gb) and 2,189,971 paired-end reads with a length of 151 bp (0.66 Gb) were obtained from the sequencing, and 88% of the bases had a quality score of >Q30. As a result, 7,609,281 clean paired-end reads (3.26 Gb) were assembled into 46 contigs with 433-fold coverage. The contig *N*_50_ value is 504,440 bp, and the largest contig is 688,257 bp. The genome size is 7.55 Mb with an average G+C content of 71.4%. A total of 6,658 protein-coding genes (1,062 of which encode hypothetical proteins), 53 tRNAs, 13 complete or partial rRNAs, 3 noncoding RNAs (ncRNAs), and 2 CRISPR arrays were predicted. As expected, the genes putatively related to steroid catabolism (aliphatic side chain degradation [*cyp125*, *fadD19*, *fadE26*, *fadE27*, *echA19*, *hsd4A*, *fadA5*, *ltp3*, *ltp4*, *fadD17*, *fadE34*, *fadE28*, *fadE29*, and *ltp2*], ring A/B [*cho*, *kstD*, *kshAB*, and *hsaABCDEFG*] and ring C/D degradation [*fadD3*, *ipdAB*, and *fadE30*], and the steroid uptake system [*mce4ABCDEF*]) were revealed in the genome of the strain.

The data from the genome-wide sequence provide precise knowledge of the *S. hirsuta* subsp. *hirsuta* VKM Ac-666^T^ gene content, which is essential for further omics studies and creation of effective biocatalysts capable of producing bioactive steroids.

### Data availability.

The raw reads have been deposited in the NCBI SRA under the accession no. SRR10222482 and SRR10222481, and the whole-genome shotgun project has been deposited in DDBJ/ENA/GenBank under the accession no. VWPH00000000. The version described in this paper is the first version, VWPH01000000. The accession number of the 16S rRNA gene sequence deposited in DDBJ/ENA/GenBank is MN515057.
